# Evaluation of Viscosity Dependence of the Critical Meniscus Height with Optical Fiber Sensors

**DOI:** 10.3390/s21238130

**Published:** 2021-12-05

**Authors:** Leonardo Binetti, Fraser Simpson, Lourdes S. M. Alwis

**Affiliations:** School of Engineering and the Built Environment, Edinburgh Napier University, Edinburgh EH10 5DT, UK; l.binetti@napier.ac.uk (L.B.); 40295738@live.napier.ac.uk (F.S.)

**Keywords:** critical meniscus height, dynamic viscosity, spectroscopy, surface tension

## Abstract

Conventional means of data extraction using optical fiber interrogators are not adequate for fast-paced detection of a target parameter. In this instance, the relationship between the critical meniscus heights (CMH) of several liquids to the extraction speed of a rod submerged in them, have been analyzed. A limitation of a previous interrogator used for the purpose had been light absorption by the liquid due to the used bandwidth of the readily-available light source, i.e., C-band. The newly proposed technique addresses this limitation by utilizing a broadband light source instead, with a Si-photodetector and an Arduino. In addition, the Arduino is capable of extracting data at a relatively faster rate with respect to the conventional optical interrogator. The use of a different operational wavelength (850 nm instead of 1550 nm) increased the r^2^ and the sensitivity of the sensor. The new setup can measure surface chemistry properties, with the advantage of being comparatively cheaper than the conventionally available interrogator units, thereby providing a suitable alternative to conventional measurement techniques of liquid surface properties, while reducing material waste, i.e., in terms of the required volume for detection of a target parameter, through the use of optical fiber.

## 1. Introduction

The recent outbreak of COVID-19 has brought forward many important improvements in biomedical and chemical engineering, at a seemingly impossible pace. One of the most notable developments has been the polymerase chain reaction (PCR) self-test kits containing a swab, a test stripe and a small amount of liquid. These have been manufactured, posted, collected and the results analyzed, within a matter of days. The most important part of the kit is the small vial, containing the liquid. From the COVID-19 predicament and from a production perspective, what is apparent is the importance of identifying the least amount of liquid that can “do the job”. This is important because even a small increment of liquid volume per vial, i.e., an increment of even 1 mL, would be considerable given mass production and, therefore, the volume of liquid needed for adequate detection. This recent example, therefore, demonstrates the importance of utilizing techniques that can reduce chemical waste, which would have a direct impact on the economic and environmental aspects of these chemicals, i.e., slightly more volume would require a larger container, which would mean more plastic material to be used for the container, etc.

The chemical properties of a liquid, i.e., one intended for the PCR kit, play a vital role in identifying the quality factor of a chemical and determining whether it meets the pharmaceutical requirements or in identifying biological signatures, i.e., for medical diagnosis. The question then would be identifying the optimal amount of liquid required for a target purpose, in the interest of reducing waste and limitations in the availability, i.e., blood/urine sample. In this case, the detection mechanism would have a direct impact on the volume of liquid/chemical needed for successful, and ideal fast-paced, diagnosis. The time factor is, therefore, as important as the other factors considered here. For example, the quality of many biomedical liquids decreases with time [1,2,3], which changes their surface chemistry properties. Therefore, effective measurement of physical-chemical properties of a liquid utilizing the least amount of liquid and a fast-paced technique, is in considerable demand in the modern era.

The surface chemistry properties of a liquid provide useful information on the physical-chemical properties of a given liquid. For example, measurement of contact angle is used in clinical settings, i.e., the contact angle of water and saliva on complete denture plastics relates to the retention of the denture [4]. In medical diagnostics, the surface chemistry properties of a liquid can be utilized to diagnose diabetes [5]. The recent technological interest and advances in medical diagnosis use a smartphone [6] to support further the case for new thinking in the direction of minimal samples [7]. Therefore, it is of great interest to explore whether the techniques used for the measurement of surface chemistry properties of a liquid can be optimized [8].

One of the most widely used conventional techniques to measure the surface properties (like surface tension and contact angle) of a liquid is the Wilhelmy method [9,10,11,12,13,14,15,16,17,18,19]. This method is based on measuring the contact angle of a liquid with respect to a solid when the surface tension of the liquid is known. The contact angle will show good or bad wetting properties of liquids if it is less than 90° or higher than 90°, respectively [20]. The plate used in this method is made out of platinum which is usually roughened to increase its surface wettability. In addition, the plate is also pre-wetted in the liquid under analysis to have a zero-contact angle. However, this technique is relatively more expensive, and the wastage of chemicals is quite high, i.e., typically 40 mL used for each analysis. In addition, because of the high surface energy of the platinum plate used in the Wilhelmy method, this method cannot be used to measure the interfacial surface tension of liquids, often found in biomedical applications [10]. In addition, the Wilhelmy plate method is limited when the viscous relaxation is too long, which affects the contact angle measurement of liquids [21].

Silica optical fibers have been used to decrease the amount of liquids, as shown by previous work, where the critical meniscus height (*CMH*) was used to measure the contact angle on the silica fiber, for small volume liquids [22]. The *CMH* represents the maximum height that the liquid meniscus reaches before collapsing, when pinned at the edge of the solid (e.g., optical fiber), extracted from the liquid. The use of fiber instead of a plate gives considerable advantages. For example, steady-state measurements are possible with high viscosity liquids, which is a challenge at present [23]. The meniscus height rapidly settles at equilibrium on small diameter and stationary fiber, resulting in high accuracy of results [24]. This had been achieved due to the considerably small cross sectional-area exposure demanded by optical fiber, i.e., 125 μm diameter. Other advantages of using optical fiber for the purpose are its durability and electrical passivity, both of which are significant aspects considered in medical/chemical equipment. However, the utilization of optical fiber still remains quite expensive due to the expense of typical laser interrogator units [25,26,27]. In addition, the standard wavelength used by the interrogator (1550 nm) [28,29,30] is absorbed by some liquids, such as water. This increases the liquid temperature. This, in turn, affects the surface chemistry properties of water, i.e., surface tension and contact angle, which change with temperature [31,32,33,34]. Therefore, a suitable interrogator which targets the required detection wavelength range must be carefully chosen or implemented.

One important aspect of bio-liquid measurement is the detection speed, and this would depend on the physical-chemical properties of the liquid and the detection technique. For example, the contact angle, i.e., between the fiber and the liquid, varies with speed, especially if the threshold between the static and dynamic speed range (20 mm/min) is exceeded [35]. At higher speeds, the contact angle will depend on the viscosity of the liquid [36]. Therefore, when evaluating the rate of submergence and extraction on the surface chemistry properties, the detection system should have a sufficient rate for data extraction in order to minimize the loss of data at crucial points. Faster data extraction also becomes crucial with particular bio-liquids that expire within a short time.

The paper presented here compares variations in *CMH* using two interrogator setups (a new interrogator setup and the current standard optical fiber interrogator unit). This new setup involves a relatively less expensive light source (LED halogen source) compared to the laser interrogator unit. In addition, the new setup uses a faster and cheaper recording rate device, such an Arduino (~130 Hz), which increases the data recording by more than 60 times compared to the laser interrogator (2 Hz). Moreover, the new setup uses a less expensive wavelength filter, by implementing a fiber coupler centered at the wavelength that is not absorbed by the liquids under analysis. Therefore, with the new interrogation setup, the *CMH* of liquid can be analyzed at a wavelength that is not absorbed by the liquid, in a shorter amount of time, and using a cheaper and easy to implement setup. In addition, using the proposed scheme, the *CMH* is evaluated to measure surface tension and dynamic viscosity of different liquids at room temperature.

## 2. Materials and Methods

### 2.1. Chemicals

The following chemicals used were of analytical grade, with no other purifications being carried out. Isopropanol, 99.7% in water (IPA), acetone 99%, glycerol ≥99.5%, diiodomethane 99%, ethylene glycol ≥99%, benzene, and paraffin oil were purchased from Sigma Aldrich Co. Ltd. (Irvine, Scotland, UK). Extra pure, deionized water was obtained from Acros Organics. P3 mineral oil with a density of 870 kg/m^3^ at 20 °C, was purchased from Pfeiffer Vacuum. Engine oil 5W-40 was bought from Anton Paar 854 kg/m^3^ at 20 °C. In this work, the single mode silica optical fiber used was of standard grade (ITU-T G.652.D).

### 2.2. Critical Meniscus Height and Surface Tension Dependence

As shown in [22], the process of measuring the critical meniscus height is accomplished by firstly attaching the optical fiber vertically to a support. A beaker containing the solution is moved up and downwards using a tensile machine (Lloyd Instruments QA LRX 05). When the cleaved optical fiber is connected to the laser interrogator (Micron Optics sm125), the reflected optical power output can be read by a computer connected to it. Moreover, a different reflected optical power is recorded when the fiber is in the air or in contact with the liquid. Before the measurement starts, the liquid is placed close to the fiber-end (e.g., 1 mm) and then moved at a constant speed until the fiber registers the reflected optical power variation due to the liquid. Thus, once the fiber is in the liquid, the optical power varies and the submergence is made to stop [22]. When the 125 μm diameter optical fiber is in the process of being extracted from the specific liquid sample, it reaches a maximum (maxima of Harkins [37]), which occurs when the meniscus volume reaches its maximum value by pinning at the edge [38] of the cleaved optical fiber. Furthermore, the meniscus decreases its volume and eventually breaks. As mentioned before, the maximum height just before the breakage of the meniscus represents the *CMH*. When that occurs, an optical power variation is observed due to the different refractive index of the liquid compared to air. Also, the liquid surface returns to its undisturbed state, i.e., flat surface, and the optical fiber registers a reflected amplitude interference since a Fabry–Perot cavity is formed between the fiber end and the flat liquid surface.

The liquids in Table 1, present surface tension, density, and viscosity values of several carefully selected liquids, whose characteristics are comparable to that of bio-liquids such as blood, i.e., for biomedical usage. However, unlike expensive bio-liquids, these chosen liquids are readily available and are cost effective, thus suitable for the laboratory work conducted herein. Also, since the optical fiber radius (*R*) is of 62.5 μm, the Bond number (β^2^ = ρgR^2^/γ) is smaller than 0.1 (where *g* is the acceleration due to gravity, ρ is the density of the liquid, and γ is the surface tension of the liquid), the density values of liquids would not affect the *CMH* measurement. In addition, the speed of motion of the fiber is 0.1 mm/min, therefore the viscosity effect is not predominant. Thus, only the surface tension is the measured quantity here to appreciate the variation of the *CMH*.

### 2.3. Critical Meniscus Height and Viscosity Dependence

In addition to the variation of the *CMH* with the surface tension, a variation of critical meniscus height due to the dynamic viscosity values, is expected, as shown in [39]. Table 2 presents six liquids, which can be grouped into three groups with two liquids each (Acetone-IPA, Benzene-P3 Oil, Paraffin-Engine Oil). The liquids in each group present the same density and surface tension but different values of dynamic viscosity. Thus, the *CMH* variation is seen between the lowest and the highest liquid dynamic viscosity within their group. In addition, the speed of extraction varies from 5 to 100 mm/min, so that the variation of the *CMH* is also seen in the transaction between the static and dynamic regions.

As seen in Table 2, some negligible differences in density values still exist due to the challenge of finding readily available liquids with the same properties. As mentioned in the previous section, the small fiber radius makes the Bond number negligible, as shown in Table 2. Therefore, the density variations between two liquids of the same group would not affect the *CMH* measurement. However, the importance of dynamic viscosity (*μ*) over the surface tension forces of the liquid is better represented by the Ohnesorge number (Oh = μ/(ργR)), which is typically used to measure the influence of the viscosity forces over the inertial and surface tension forces. This is illustrated in Table 2.

### 2.4. Spectroscopy

As mentioned in the introduction, the properties of water and other liquids change with temperature. The absorption of radiations could induce temperature variations at particular wavelengths. For example, water absorbs in the infrared region. Thus, all liquids were analyzed with PerkinElmer UV/VIS/NIR Spectrometer Lambda 750. In the spectrometer, liquids are covered to avoid any unwanted external radiation reaching them. For this reason, as in the spectrometer, the container of the liquids under analysis for measuring the *CMH* was covered with Tin foil to avoid any unwanted wavelength radiation reaching the liquid during the analysis.

### 2.5. Light Source and Filters

As mentioned in the previous section, the possible operable wavelength “window” must be chosen so that the liquids do not absorb radiations. Thus, a tungsten halogen light source (HL-2000) from Ocean Optics was used for the work, presenting a wider wavelength band, i.e., 360–2400 nm, compared to the interrogator. In addition, the expensiveness of interrogation is reduced by replacing the laser interrogator unit with a relatively cheaper light source. However, this halogen source presents a broader range that must be narrowed down so that the injected radiation wavelength is not within the absorbing region of the liquids. In other words, only the wavelengths corresponding to radiations that are not absorbed by liquids, are used.

The experiment uses an optical coupler (“Y” shaped 50:50 coupler) instead of a circulator since a circulator is more expensive. The measurement of the optical power output transmitted into the fiber is analyzed with an optical spectrum analyzer (OSA) —(Yokogawa AQ6370D-02-LI-Q/FC/RFC)—from 600 to 1700 nm. The transmitted radiation is then injected into the liquid.

**Figure 1:**
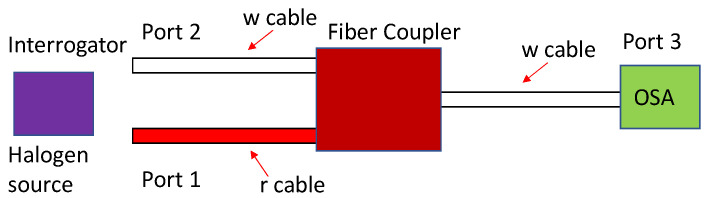
Depiction of the fiber coupler design when the interrogator and halogen light source is connected to either Port 1 or 2 while the optical spectrum analyzer (OSA) detects the transmitted signal.

The coupler, shown in Figure 1, presents three optical fiber ports. Port 3 (white cable, w) was connected to the OSA while the light source was connected to one of the two remaining ports progressively, and the OSA read the output value. In this experiment section, two couplers were used. The first was an 850 nm coupler (TW850R5F1 from Thorlabs), in which the fiber used in this coupler was the relatively inexpensive 780-HP type, optimized in the near-infrared wavelength (in the wavelength region of 780–970 nm as suggested by the manufacturer). In addition, this fiber has a smaller core compared to the standard optical fibers (SMF-28) 4.4 and 8.2 μm, respectively. The smaller core dimension reduces the exposure of the liquid to the light transmitted by the optical fiber and the exposure of the liquid to the optical core material, which is different from the optical fiber cladding material. However, both fibers (SMF-28 and 780-HP) have the same cladding dimension of 125 μm. The second coupler had its wavelength centered at 1550 nm (TW1550R5F1 from Thorlabs). In addition, in terms of comparison, both the interrogator and the halogen source were used to see the filtration of the optical power signal transmitted through the fiber to the OSA.

### 2.6. Connection and Data Recording

Once the chosen light source and coupler configuration had been agreed, the remaining Port was then connected to a Si-photodetector (DET02AFC from Thorlabs, Newton, NJ, USA) and then connected to an Arduino (Uno), which is an inexpensive data logger. The data from the Arduino was then read by a computer at the rate of ~130 Hz, which is more than 60 times faster than the recording rate of the current laser interrogator unit (2 Hz).

## 3. Results and Discussion

### 3.1. Critical Meniscus Height (CMH) and γ Dependence

**Figure 2:**
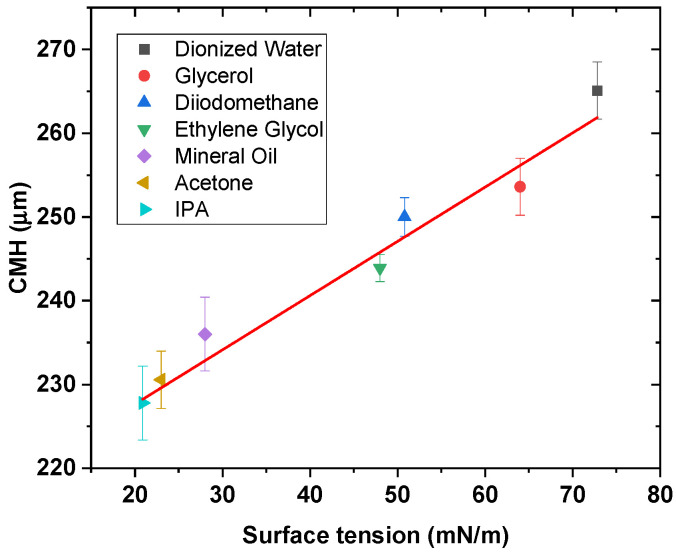
Depiction of the critical meniscus height (*CMH*) in response to variation in the surface tension of the liquid.

As mentioned in method Section 2.2, the *CMH* is measured for different surface tension liquids in Table 1. The speed of extraction of the fiber was 0.1 mm/min. At this speed, glycerol, which presents the highest value of the dynamic viscosity *μ* among the solutions chosen, presents a negligible impact on the surface tension properties, as shown in [35]. In addition, because of the cleave present on the fiber-end and its cylindrical shape, it could be assumed that no “end-effect” would be present at the fiber-end, as shown in [22]. A difference of the *CMH* as the surface tension of the liquid changes is observed, as presented in Figure 2, with a gradient of 0.65 ± 0.07 μm/(mN/m) and r^2^ of 0.945. The r^2^ value demonstrates that it is not perfectly linear, and the result needs improvement. As mentioned before, some liquids may absorb light in the wavelength range selected by the optical fiber interrogator unit. This absorption may cause the liquid to increase its temperature [40]. For this reason, a spectroscopical test was required to identify the wavelength range so that the minimum wavelength absorption is registered for all liquids.

### 3.2. Spectroscopy and Light Source

**Figure 3:**
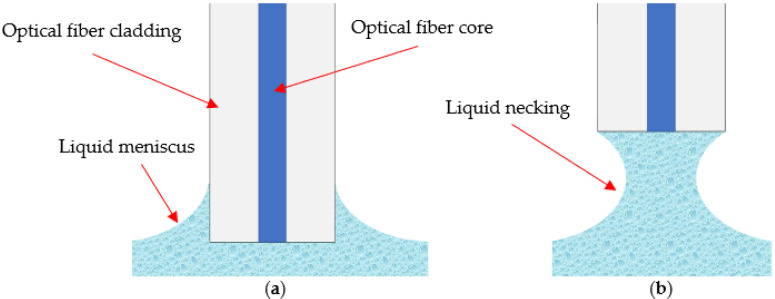
Depiction of the meniscus forming on the optical fiber (**a**), meniscus during the fiber being removed (**b**).

As mentioned in the previous paragraph, the wavelength range used by the standard interrogator unit is absorbed considerably by some liquids, and this absorption can cause an increase in the local temperature of the region where the fiber is in contact. In addition, when the fiber is about to be extracted from the liquid, the laser radiation is concentrated onto a smaller dimension of the liquid, as seen from Figure 3a to Figure 3b, which may increase the temperature of that region. The temperature increase may be more evident when the meniscus necks before the rupture, as shown in Figure 3b.

**Figure 4:**
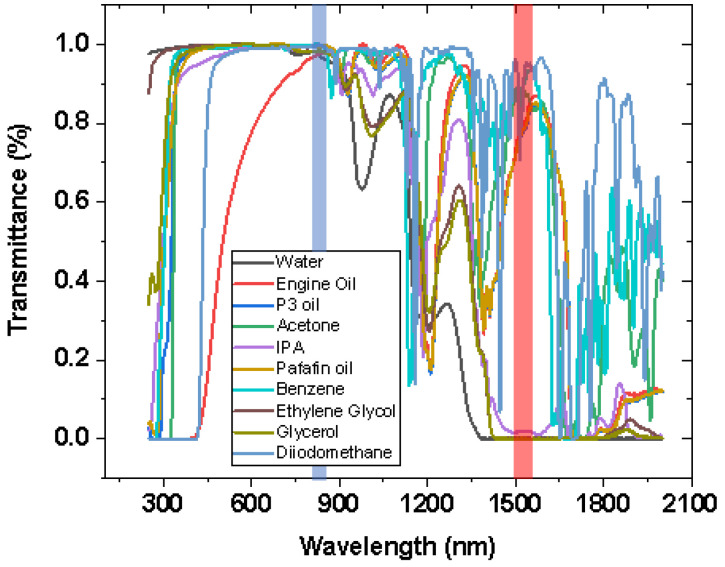
Spectroscopical analysis of the liquids present in Table 1 and Table 2. The blue highlighted section is where liquids absorb less radiation, whereas the section highlighted in red shows liquids absorbing within the wavelength range selected by the interrogator.

The spectroscopical analysis at room temperature of all liquids in Table 1 and Table 2 are presented in Figure 4. Some liquids like water, IPA, glycerol, and ethylene glycol, present a relatively lower transmission rate in the wavelength range used by the interrogator. In other words, those liquids absorb in the communication C-band (highlighted in red). However, as shown in Figure 4, there is a wavelength range where all the liquids absorb less. This region is within the wavelength range between 800 and 850 nm (highlighted in blue). For this reason, the light source must be changed, as shown in the next section.

### 3.3. Light Source and Filters

As mentioned in Section 2.5, the wavelength that reaches the liquid must be changed/filtered, so that the liquid does not absorb the wavelength. Therefore, as mentioned before, the two light sources (halogen and the laser interrogator unit) are compared and connected to two couplers (850 nm and 1550 nm wavelength centered couplers), in order to filter the right wavelength.

**Figure 5:**
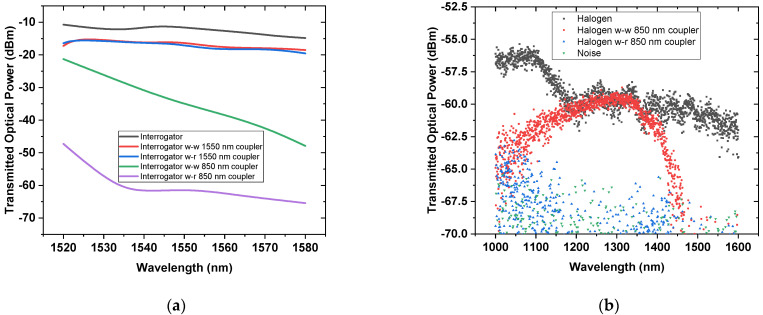
Transmission optical power via optical fiber coupler when the interrogator (**a**) or the halogen light source (**b**) is used.

Figure 5a,b shows the results of the transmitted optical power of the interrogator and the halogen light source, respectively. Figure 5a shows the transmitted optical power (registered by the OSA), when connected directly to the interrogator. Here, the signal is only affected in the range between 1520 and 1580 nm, as expected. In this range, the interrogator presents a transmitted optical power equal to ~–10 dBm. A negligible difference between Port 1 and 2 is seen if the coupler at 1550 nm is used since the wavelength radiation of the interrogator matches the wavelength transmittable by the coupler used.

In conclusion, no filtration of the interrogator signal is presented using this coupler. However, different values of transmitted optical power are registered when the interrogator is connected to the coupler centered at 850 nm. In fact, a difference is seen if Port 1 (red cable, r) or Port 2 (white cable, w) is used. In the first case, the transmitted optical power is between –40 and –65 dBm, whereas the second is between –20 and –50 dBm, in the range between 1520 and 1580 nm. The reduction in the transmitted optical power means that the optical fiber has absorbed radiation. Therefore, this radiation is not absorbed by the liquid, and the coupler acts as a filter.

In Figure 5b is shown that the transmitted optical power of the light source is weaker than the interrogator, ~–60 and ~–10 dBm, respectively. Therefore, the impact of possible optical power variation [41] on the liquid is negligible at ~–60 dBm. In addition, to avoid any output instability, it was made sure that the fiber was not overly bent, and the experiment was conducted at room temperature. However, the weaker signal is expected throughout the entire range where liquids absorb the most. In addition to the weaker signal, in this case, the optical fiber absorbs less power intensity. Therefore, a negligible temperature rise could be present in the fiber.

Led by the conclusion from the previous paragraph, the halogen light source was connected to Port 1 and 2 (of the 850 nm coupler). Some transmitted optical power is still present when the light source is connected to Port 2. A different scenario is present if the same light source is connected to Port 1. In this case, the transmitted optical power decreases below the thermal noise. For this reason, this configuration is used for sending light to the liquid through Port 3. Thus, Port 2 was connected to the Arduino. In addition, the wavelength at 850 nm is not absorbed by the liquids, as shown in the section highlighted in blue in Figure 4.

### 3.4. Connection and Data Recording

**Figure 6:**
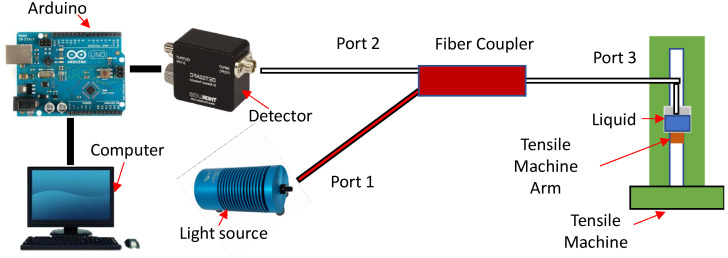
Depiction of setup used for measuring the *CMH* of a liquid with a different light source and the detector connected to the Arduino. A computer then reads the signal.

Once the chosen light source (halogen) and coupler (850 nm centered wavelength) configuration had been agreed, the remaining Port 2 was connected to the Arduino, as shown in Figure 6.

**Figure 7:**
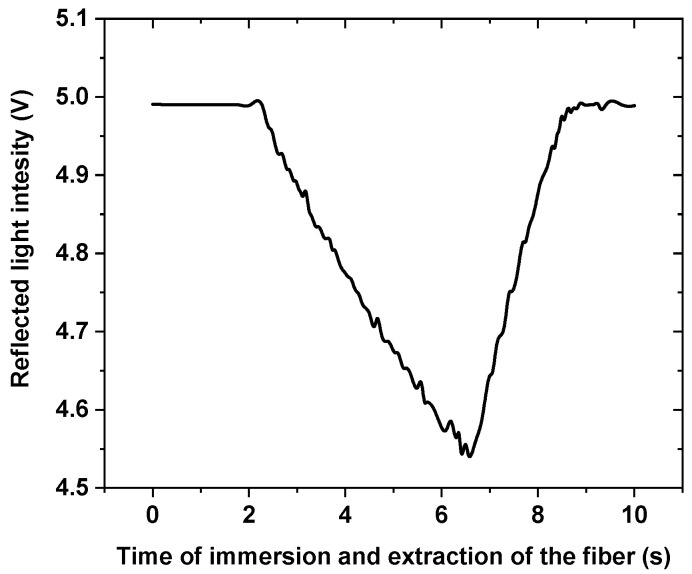
Depiction of the voltage signal provided by the Arduino while the detector registers the voltage variation recorded in the process of immersing the fiber and removing the fiber from one of the selected liquids (P3 oil in this case).

A generic experimental result is presented in Figure 7, which shows the output signal coming from Port 3. The fiber is in air from 0 to 2 s, and a voltage closer to 5.0 V is present. From 2 to 2.3 s, the optical fiber is closer to the surface of the liquid, and the voltage increases due to an increase of reflected light coming from the leveled liquid surface. Then the voltage decreases, indicating that the optical fiber is in contact with the liquid. At this stage, the tensile machine arm is stopped. This point is considered to be the starting point to start measuring the *CMH*. In this case, a time of 4 s (from 2.3 to ~ 6.3 s) is allowed for the fiber to register an optical power variation. The fiber is then extracted from the liquid at a constant speed until the meniscus breaks. During this time, the measured voltage will continue to decrease, and once the meniscus breaks, the voltage will increase. The time duration from the start of the motion of the optical fiber to the time of registering an increase in the voltage, is recorded. This time is then translated to length (e.g., *CMH*) using the speed of motion. Therefore, the voltage-time curve in Figure 7 will change this time difference for each liquid, since they present different *CMH* values. However, the signal continues to increase until a plateau is reached at a time of ~8.7 s. In addition, a voltage difference is present between the voltage registered at the start of the measurement and that measured at the end. This is due to residual liquid present at the end of the fiber. For this reason, the optical fiber was cleaned with a detergent, acetone, and then with water and before being dried in air until the signal was restored to one of the cleaved fibers in the air.

Validation of the current method for measuring the *CMH* of the liquid has been conducted and discussed in the next section.

### 3.5. Validation and Differences between Two Methods

To validate the new setup for measuring the *CMH* present in Figure 6, some authors’ previous work [22] was considered. In this case, the P3 mineral oil was considered since it does not absorb in the selected wavelength range (850 nm), as shown in Figure 4. The result shown in this validation confirmed the value obtained at the same speed of 0.1 mm/min, previously analyzed by [22]. However, a discrepancy of approximately 10 μm was noticed when water was used. This difference is possible since the water strongly absorbs the wavelength range of the interrogator unit, presenting a strong optical power emission in that range.

By contrast, water absorbs less in the range selected by the optical coupler, and the optical power of the light source is considerably less compared to the interrogator. In addition, there were some differences compared to the results obtained in Figure 2, using the interrogator. Liquids like glycerol, ethylene glycol, and IPA presented an increased *CMH* variation of ~5, ~5, ~2 μm, respectively, compared to the results obtained previously with the interrogator. It is suspected that the difference is due to the presence of the OH^−^ groups present within the chemical structure. In addition, the correction to the evaporation rate of the liquids has decreased as the power intensity and different wavelengths were selected. Considering those values of *CMH* in the graph shown in Figure 2, the slope of the *CMH* is 0.77 ± 0.05 μm/(mN/m) with an r^2^ of 0.977. Thus, an increase of the slope and r^2^ is present in this scenario, demonstrating an improvement in the measuring method. In addition, the expense of the instrumentation has also been decreased.

### 3.6. Dependence of Speed in CMH Measurement

**Figure 8:**
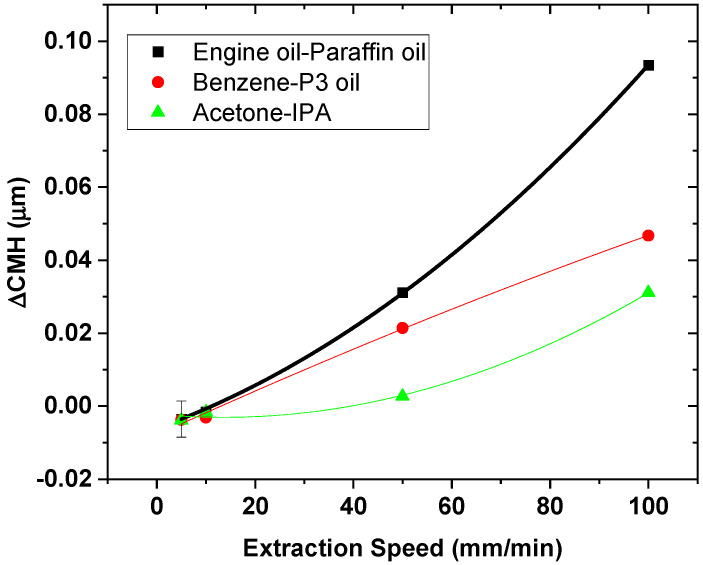
Depiction of the *CMH* variation as the dynamic viscosity of the liquid changes.

The faster recording rate of the new device and the reliable measurement of this method was essential for detecting the variation in terms of *CMH* with different dynamic viscosity liquids. As mentioned before, liquids in Table 2 present the same surface tension and density as for the three groups of liquids, such as acetone and IPA, benzene and P3 oil, engine and paraffin oil. However, they present different dynamic viscosity values at room temperature. Figure 8 shows the normalized difference between the three groups of liquids, as mentioned in the method Section 2.3. The graph shows that the increase of the *Oh* number increases the difference in terms of the critical meniscus height as the speed of the fiber increases. This trend is comparable to the data shown in [39]. However, there is less variation of the *CMH* between the three groups at a lower speed than higher speed. In addition, as soon as the speed increases over the threshold between static and dynamic, the variation in *CMH* between the three groups increases as the speed increases.

## 4. Conclusions

The classic optical interrogators are bulky, need specialized skills for operation, and are expensive. In addition, the data extraction speeds are not sufficient for a fast-paced detection, especially with respect to measurement of physical-chemical properties of liquids. The paper presented here presents a new, cost-effective, fast, and reliable optical fiber-based sensor technique to measure the *CMH* of a wide range of liquids. In addition, this method was compared to the results obtained by the laser interrogator unit from a previous study [22]. The result showed a variation in *CMH* values for some liquids, and this could be attributed to their high absorbance in particular wavelength ranges due to the high-power-laser light source, as opposed to variations in the measurand. In addition, a fiber coupler was used as a filter, so that only the target wavelengths, i.e., wavelengths that are not absorbed by the liquids, were injected in the liquid. Thus, the liquids did not absorb the wavelength, which means the results were not influenced by temperature fluctuations induced by wavelength absorption. Furthermore, the speed of removing the fiber from the liquid provided a variation of the *CMH*. This value was approximately the same for low speeds, whereas it increased as the speed went beyond 20 mm/min. The proposed system showcases a time- and cost- effective solution for the measurement of physical-chemical properties in liquids, compared to state-of-the-art methods.

## Figures and Tables

**Table 1:** Surface tension.

Liquid	Density (kg/m^3^)	Dynamic Viscosity (mPa·s)	Bond Number (10^−2^)	Surface Tension (mN/m)
Deionized Water	997	0.89	2.3	72.8
Glycerol	1126	950	2.5	64.0
Diiodomethane	3320	2.6	5.0	50.8
Ethylene Glycol	1097	16.2	3.0	48.0
Mineral Oil	870	82.65	3.5	28.0
Acetone	791	0.3	3.6	23.0
IPA	785	2.04	3.8	20.9

**Table 2:** Liquid properties.

Liquid	Density (kg/m^3^)	Surface Tension (mN/m)	Dynamic Viscosity (mPa·s)	Bond Number (10^−2^)	Ohnesorge Number
Acetone	791	23	0.3	3.6	0.009
IPA	785	21	2.04	3.8	0.063
Benzene	870	28	0.603	3.5	0.015
P3 Oil	870	28	82.65	3.5	2.118
Paraffin Oil	827	26	140	3.5	3.818
Engine Oil	854	25	206	3.6	5.639
